# Initial psychometric evaluation of the Portuguese version of the Liverpool Osteoarthritis in Dogs

**DOI:** 10.1186/s12917-022-03461-8

**Published:** 2022-10-06

**Authors:** J. C. Alves, P. Jorge, A. Santos

**Affiliations:** 1 Guarda Nacional Republicana (Portuguese Gendarmerie), Lisbon, Portugal; 2 Instituto de Investigação e Formação Avançada, MED – Mediterranean Institute for Agriculture, Environment and Development, Universidade de Évora, Pólo da Mitra, Ap. 94, 7006-554 Évora, Portugal

**Keywords:** Dog, Osteoarthritis, Hip, Liverpool Osteoarthritis in Dogs, Clinical Metrology Instruments

## Abstract

**Background:**

Osteoarthritis (OA) is the most commonly diagnosed joint disease in companion animals, and proper tools are necessary to assess patients and response to treatment. We aimed to perform an initial psychometric evaluation of the Portuguese version of the Liverpool Osteoarthritis in Dogs (LOAD).Fifty Police working dogs with bilateral hip OA were assessed in a prospective, randomized, double-blinded study. Canine handlers, who were native Portuguese speakers, completed a copy of the translated version of the LOAD. Their results were compared with those of fifty sound dogs. Construct validity was evaluated by assessing differences between OA and sound animals with the Mann-Whitney test. Further evaluation was performed with the Kaiser-Meyer-Olkin measure of sampling adequacy, Eigenvalue, and scree-plot analysis. Internal consistency was tested with Cronbach’s α.

**Results:**

A significant difference was observed between OA and sound dogs (p < 0.01), indicating construct validity. Two factors accounted for 81.5% of the total variance. Cronbach’s α was 0.96, and a high inter-item correlation was observed, raging from 0.76 to 0.95, showing strong internal consistency. We presented criterion and construct validity of the Portuguese version of the LOAD, which is valid for use in the Portuguese language. It is an additional stage in providing a broader number of clinicians with an accessible tool to evaluate dogs with osteoarthritis. Further studies are required.

## Background

Osteoarthritis (OA) is the most commonly diagnosed joint disease in veterinary medicine. It has a toll on patients’ quality of life, implying a considerable cost in healthcare[1, 2]. Having clinically relevant outcome measures is paramount to evaluating patients and determining response to treatment[3]. For that purpose, different clinical metrology instruments have been developed to measure pain and impairment in performing daily activities. This patient-centered approach has been incorporated into veterinary assessments[4–6]. A clinical metrology instrument comprises a sequence of questions or items, scored based on the person’s completing it observations or experiences. The individual item scores are then used to calculate an overall instrument score[7]. They may also present an alternative or complement to objective measures, as a change in load-bearing of an individual limb may not be correlated to a change in demeanor or activity in the animal’s everyday environment[7, 8]. Similarly, an increased joint range of motion may not be significant if the patient shows no improvement in its ability to perform daily activities[9].

The Liverpool Osteoarthritis in dogs (LOAD) was initially developed to assess dogs with elbow OA. It has shown good reliability, just lower than peak vertical force generated by force plate gait analysis, although both results correlate[7, 10]. Later, its broader use has been tested and is deemed reliable to assess canine OA in general[7]. The development of clinical metrology instruments has been extensively documented. If an instrument is translated, several properties must be assessed in the target population after translating the instrument to the desired language[3]. Validity is determined through different approaches. Face validity is judged by a group of experts that assess if the scale looks reasonable for the purpose set. Construct validity is evaluated when the target attribute cannot be observed directly[11]. Factor analysis is usually used to assess construct validity, and Cronbach’s α allows to assess internal consistency[5, 7, 12]. In addition, the instrument’s reliability must be determined to assess if the questionnaire is delivering consistent results[11].

The goal of this study was to validate a Portuguese version of the LOAD, allowing its use in studies where the target population has Portuguese as a primary language, spoken by 261 million people around the world[13]. We hypothesized that the Portuguese version would show the reliability and validity documented in the English version.

## Results

The sample included 100 Police working dogs, of both sexes (55 males − 30 OA and 25 sound dogs, and 45 females − 24 OA and 21 sound dogs), with a mean age of 7.4 ± 3.2 years and a bodyweight of 24.1 ± 7.2 kg. Four breeds were represented: German Shepherd Dogs (n = 34, 18 OA and 16 sound dogs), Belgian Malinois Shepherd Dogs (n = 30, 12 OA and 18 sound dogs), Labrador Retriever (n = 20, 10 OA and 10 sound dogs), and Dutch Shepherd Dog (n = 16, 9 OA and 7 sound dogs).

**Fig. 1:**
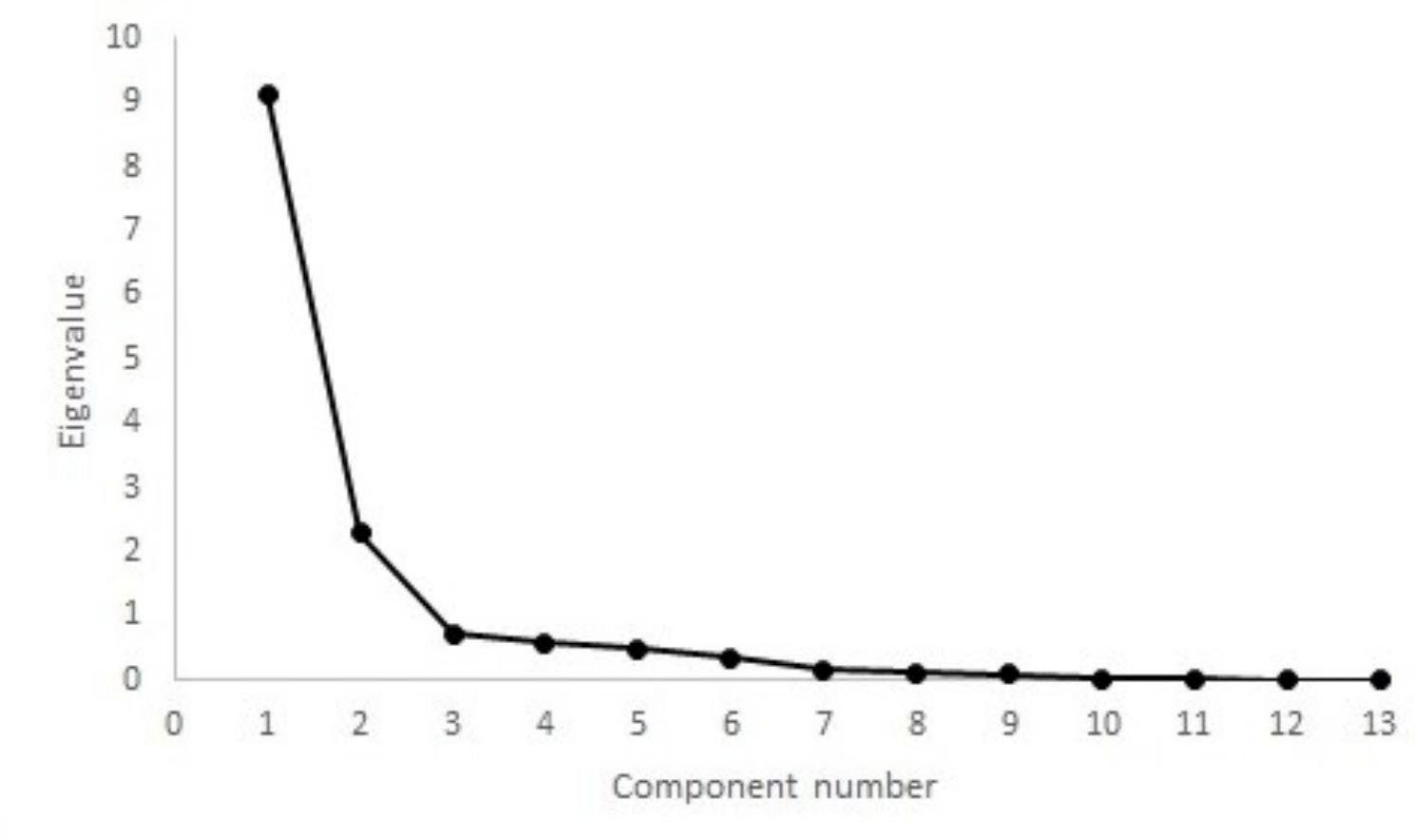
Scree plot of factor analysis of the Portuguese version of the Liverpool Osteoarthritis in Dogs. Two factors had Eigenvalues > 1, with a discernible “shoulder” observed

A significant difference was observed between OA and sound dogs (p < 0.01), with sound dogs showing lower scores (median 8.0, interquartile range 5.0) than OA dogs (median 22.0, interquartile range 14.0). Cronbach’s α, measuring internal consistency of the test items, was 0.96. The Kaiser-Meyer-Olkin measure of sampling adequacy was 0.95. As all values were above 0.8, factor analysis was conducted. The varimax-rotated model of factor analysis identified two factors with an eigenvalue > 1, accounting for 81.5% of the variance (65.2% and 16.3%, respectively). The remaining factors have eigenvalues < 0.8. A scree-plot (Fig. 1) confirmed the retention of the two factors.

Based on the varimax-rotated solution, loading for these two items was performed. Loading values > 0.4 indicate good correlation of the item with the factor[14]. All items loaded heavily on the first component, with commonalities ranging between 0.67 and 0.92. A communality value < 0.40 may indicate that the item is not related to the other items in that factor[14]. Table 1 summarizes the loading for items on each of the two extracted factors. A high inter-item correlation was observed, raging from 0.76 to 0.95.

**Table 1:** Item loading for components extracted by factor analysis of LOAD, based on varimax rotated solution

Factor	Item		Factor loading	Communality
1	5	Rigidez após descanso	0.90	0.86
	2	Incapacidade causada pelo coxear	0.88	0.86
	13	Efeito do coxear na capacidade de se exercitar	0.88	0.79
	12	Rigidez após exercício e descanso	0.87	0.79
	1	Mobilidade geral	0.85	0.75
	8	Capacidade em exercitar	0.81	0.78
	10	Frequência de descanso durante o exercício	0.81	0.67
	3	Nível de atividade geral	0.69	0.92
	7	Gosto em se exercitar	0.68	0.88
	6	Nível de atividade durante o exercício	0.65	0.87
	9	Efeito do exercício no coxear	0.74	0.71
2	11	Efeito do tempo na capacidade de se exercitar	0.82	0.78
	4	Efeito do tempo no coxear	0.64	0.74

## Discussion

The LOAD translation to Portuguese is an additional and essential step in broadening the availability and use of this validated instrument in daily practice and research. It also allows for comparing results between studies and multination cooperation in international studies[11]. This study shows that the Portuguese version of the LOAD has an adequate internal consistency and construct validity in a group of dogs with hip OA, similar to what has been described before for the original English version[7].

The evaluation of instrument validity provides evidence that it measures what it is supposed to measure[7, 12]. Construct validity can be assessed through factor analysis, and internal consistency is most frequently tested using Cronbach’s α [5, 7, 12]. Our results for factor analysis extracted a different number of components compared with previous reports (2, in contrast with the described 3)[7]. However, different factor analysis results are not uncommon for different populations. Specifically, we have to keep in mind the nature of this study’s population, which is composed of a relatively homogeneous set of breeds, similar in size and conformation. In addition, all animals had OA of the same joint, bilateral in all cases, and experienced a comparable activity level. It may also be attributed to the different persons’ variable ability to complete the LOAD in detecting the clinical signs[15, 16]. This version of the LOAD was completed by the dogs’ handlers, which are used to observing working dogs and are sensible in detecting changes in dogs, particularly their own. On the one hand, a pet owner may be less aware of these changes, but on the other hand, with proper education and due to the extended amount of time they share with their pet, they should be able to detect these changes.

We also performed an alternative measure of construct validity through factor analysis. Two factors were extracted with eigenvalues greater than one and through scree-plot analysis. Item loading of the components for LOAD identified items that could be described with “ability to exercise” and “effect of weather”. Factor loading was also supported by the good inter-item correlations and Cronbach’s α[11]. While Pearson correlation has been found to underestimate the strength of relationships between items[17], it should not be a problem as correlation values were high. And while our results validate the Portuguese version of the LOAD, its properties should evaluate with an objective measure as a comparison. In addition, the study population is very homogenous, and all dogs had bilateral disease of the hip joint only. For that reason, future studies should include a larger number of patients with heterogeneous characteristics. While the English version of the LOAD has been able to evaluate response to treatment[7, 10], the responsiveness of the Portuguese version needs to be determined. Still, we presented enough data that shows that the Portuguese version of the LOAD addresses the clinical manifestations of OA and can differentiate sound from OA dogs.

## Conclusion

In this study, we determined the criterion and construct validity of the Portuguese version of the LOAD and that it is valid for use in the Portuguese language. Further studies are required to determine if the present results can be replicated across samples with different characteristics and evaluate response to treatment.

## Methods

Permission to translate the LOAD into Portuguese was obtained from the copyright holder, Elanco Animal Health. The English version was translated into Portuguese by a group of veterinary experts, fluent in the target language. This version was then backward translated into the original language by a bilingual reviewer[3, 11, 18]. The LOAD is composed of thirteen items, and the response to each question corresponds to a value ranging from 0 to 4, where 0 represents a healthy animal and 4 a case of severe disease. The sum of all questions’ values renders the final instrument score[10]. The English version of the LOAD is available online (https://dspace.uevora.pt/rdpc/bitstream/10174/19611/2/liverpool%20OA%20in%20dogs%20-%20load.pdf). A full copy of the Portuguese version of the LOAD is also available online (http://vetpt.columbus.acsitefactory.com/sites/g/files/adhwdz991/files/2021-09/PTCACONS00002%281%29_LOAD ONSIOR.pdf).

A sample of 100 police working dogs of both sexes was used, constituting a convenience sample. Fifty patients had bilateral hip OA, and 50 were sound dogs. The diagnosis of bilateral hip OA was based on history (difficulty rising, jumping, and maintaining obedience positions, stiffness, and decreased overall performance), physical examination (pain during joint mobilization, stiffness, and reduced range of motion), and radiographic findings consisting with painful appendicular osteoarthritis[11, 18]. Additional inclusion criteria comprised bodyweight ≥ 20 kg, age > 2 years, and a period > 6 weeks without receiving any medication or nutritional supplements. All inclusion criteria had to be met to include the animal in the study. All animals were submitted to a physical, orthopedic, neurological examination, complete blood count, and serum biochemistry. The same researcher examined all animals. A copy of the Portuguese version of the LOAD was completed by the canine handlers[19], in a quiet room with as much time as needed to answer all items. All handlers were native Portuguese speakers.

Construct validity was evaluated by assessing differences between OA and sound animals, and the Mann-Whitney test was used. Factor analysis was performed using the Kaiser-Meyer-Olkin measure of sampling adequacy to explore the relationship between the instrument’s questions, with adequacy considered > 0.6[20]. Eigenvalue and scree-plot analysis were used to assess extracted values, and item loading on the extracted components was based on a varimax-rotated model of factor analysis. A communality cut-off value of 0.4 was considered. Correlation between items was assessed with Pearson correlationcoefficient. P-values less than 0.05 were considered significant. Internal consistency was tested with Cronbach’s α, and a value of at least 0.8 being considered reliable[3, 7, 11]. All results were analyzed with IBM SPSS Statistics version 20.

## Data Availability

All data generated or analyzed during this study are included in this published article.

## List of abbreviations

LOAD: Liverpool Osteoarthritis in Dogs.
OA: Osteoarthritis
